# Individual cultures of melanoma tumor infiltrating lymphocytes possess distinct patterns of reactivity with mutated antigens identified by whole exome sequencing of autologous tumors

**DOI:** 10.1186/2051-1426-2-S3-P8

**Published:** 2014-11-06

**Authors:** Jessica S Crystal, Yong Chen Lu, Yong Li, Mona El-Gamil, Todd Prickett, Kasia Trebska-McGowan, Cyrille Cohen, Steven A Rosenberg, Paul F Robbins

**Affiliations:** 1 National Institutes of Health/National Cancer Institute, Bethesda, MD, USA

## 

The adoptive transfer of autologous tumor infiltrating lymphocytes (TIL) can mediate the regression of tumors in up to 70% of patients with metastatic melanoma. These clinical trials have involved infusion of either bulk T cells generated by culturing cells from enzyme digests of fresh tumors in interleukin-2 (IL-2), or of T cells derived from small tumor fragments cultured in IL-2, that were subsequently expanded using the anti-CD3 antibody, OKT3, in the presence of irradiated feeder cells derived from peripheral blood. Individual fragment cultures may contain a more diverse repertoire of antigen reactivities, which may be advantageous in treating heterogeneous tumor populations. The diversity of antigen recognition by individual TIL fragment cultures has not, to date, been directly evaluated. The current study was carried out to evaluate antigen reactivity of individual fragment TIL cultures from patient 2369, who experienced a complete response to autologous TIL administration. Previous results indicated that the TIL that were administered to patient 2369 predominantly recognized two unique mutated tumor antigens. In the present study, fragment cultures were evaluated for their ability to recognize mutated candidate antigens identified by exomic sequencing encoded within tandem minigene constructs (TMGs) using recently described methods. The screening of twelve individual fragment cultures of the fresh tumor, two tumor digest cultures, and the bulk population of infused T cells for their ability to recognize TMGs encoding 162 mutated gene products resulted in the identification of two additional mutated antigens that were recognized in the context of multiple HLA restriction elements. Individual cultures recognized between zero to four of the mutated targets, and these antigens were recognized to varying degrees by individual cultures. Future studies will be directed towards evaluating TIL from additional patients for their ability to recognize mutated antigens expressed by autologous tumors in an attempt to generate cells that are more effective at mediating long-term tumor regressions.

## Consent

Written informed consent was obtained from the patient for publication of this abstract and any accompanying images. A copy of the written consent is available for review by the Editor of this journal.

